# Open science in Sámi research: Researchers' dilemmas

**DOI:** 10.3389/frma.2023.1095169

**Published:** 2023-04-13

**Authors:** Coppélie Cocq

**Affiliations:** Humlab, Umeå University, Umeå, Sweden

**Keywords:** open science, data management, ethics, indigenous research, Sámi research

## Abstract

This article discusses the challenges of Indigenous research in relation to open science, more particularly in relation to Sámi research in Sweden. Based on interviews with active scholars in the multidisciplinary field of Sámi studies, and on policy documents by Sámi organizations, this article points at the challenges that can be identified, and the practices and strategies adopted or suggested by researchers. Topics addressed include ownership, control, sensitivity and accessibility of data, the consequences of experienced limitations, the role of the historical context, and community-groundedness. This article has the ambition to contribute with a discussion about the tensions between standards of data management/open science and data sovereignty in Indigenous contexts. This is done by bringing in perspectives from Indigenous methodologies (the 4 R) and by contextualizing research practices and forms of data colonialism in relation to our contemporary context of surveillance culture. Research—in relation to ethics and social sustainability—is an arena where tensions between various agendas becomes obvious. This is illustrated in this article by researchers' dilemmas when working with open science and the advancement of Indigenous research. Efforts toward ethically valid and cultural-sensitive modes of data use are taking shape in Indigenous research, calling for an increased awareness about the topic. In the context of Sámi research, the role of academia in such a transformation is also essential.

## Introduction

Guidelines, recommendations, and laws around the process of data management have recently been changing rapidly, and universities and funding agencies are establishing standards and procedures for researchers to comply with. These are guided by principles from open science and research ethics—principles central to any field of research. In Sweden, a highly digitized society and research community, this can be observed for instance in how calls for applications and agreements in research projects are designed. Several universities are allocating financial resources, appointing experts, and developing systems in order to facilitate the implementation of data management plans, the storage of data, and the possibility of sharing research data. This is pushed by a national agenda outlined in a government bill in 2020, stating that “[t]he transition [to open science] for research data shall be fully implemented by 2026, which means that research data shall be made available as open as possible, as closed as necessary” (Government Offices of Sweden, 2020).

Within Indigenous research, the topic of data sovereignty is increasingly discussed, and general principles supporting open science are reinvigorating the debate. Ownership, control, and shareability of data are examples of issues that need to be investigated in the specific contexts of Indigenous groups, where the legacy of colonialism and of misuse of research are aspects that cannot be ignored in contemporary Indigenous research.

The purpose of this article is to discuss the challenges of Indigenous research in relation to open science. More particularly, this will be investigated in relation to Sámi research in Sweden. Based on policy documents from Sámi political bodies and organizations, as well as interviews with active scholars in the interdisciplinary field of Sámi research, this article points to the challenges that can be identified, and the practices and strategies adopted or suggested by researchers. The intention of this article is to provide a contribution discussing the tensions between standards of data management/open science and data sovereignty in Indigenous contexts.

## Research context

Sámi research is currently represented at several universities in the Nordic countries (and actually, also in other countries)^1^, as an interdisciplinary field including disciplines such as ethnology, linguistics, didactics, history, environmental sciences, medicine etc. Several of these universities offer courses from the undergraduate to graduate level.

Contemporary Sámi research builds on the foundations established by the Sámi movement in the 1970s, which strove to challenge power relations and revise the role of research and researchers in relation to Sámi communities (Keskitalo, 1994; Korhonen, 2002; Lehtola, 2017; Junka-Aikio, 2019). Such a movement emerged both as part of political efforts and as a reaction to earlier forms of research considered extractive and even harmful to Sámi communities. Similar developments in other Indigenous areas have led to a flourishing academic landscape working toward the advancement of research in relation to Indigenous groups, promoting community-groundedness and a repositioning of relationships between researcher and research participants (Tuhiwai Smith, 2008; Kovach, 2009; Chilisa, 2012). In this context, discussions on ethics as well as the ownership and control of research data are of immediate importance in the development of research and teaching with and in relation to Indigenous groups and lands.

UNESCO declared that open science has the “potential of making the scientific process more transparent, inclusive and democratic” (UNESCO, n.d.). This statement illustrates the ambitious and praiseworthy goal of open science. It is also claimed to be a necessary step toward the sustainable development goals in Agenda 2030 (UNESCO; Swedish research council for sustainable development) (FORMAS, 2021). Open science is about making research results (e.g., publications) and research data (and/or metadata) accessible to other researchers and to society. It strives for increasing not only accessibility to research, but also dialog between researchers and stakeholders, as a consequence of sharing data, results, and knowledge. The efforts for better and faster dissemination of research (from researchers to the wider society) readily embrace “the potential of digitalization for effective communication” (FORMAS, 2021).

In this context, recommendations on data management according to the FAIR principles are clearly defined by funding agencies and universities. FAIR, i.e., Findability, Accessibility, Interoperability, and Reuse of digital assets, refer to principles aiming at making research data easier to share and access. These recommendations also call attention to needs for adjustments, in that the principles “should be implemented taking into account applicable legislation, and, as far as is possible and applicable, be based on the technical, organizational and/or discipline-specific preconditions that apply” (Swedish Research Council, 2021). Indigenous research implies such “discipline-specific preconditions” that are both in line and in contradiction with open science. On the one hand, making research more transparent and inclusive, and increasing the dialog between researchers and stakeholders (e.g., local communities) is articulated as Indigenous projects (Tuhiwai Smith, 2021, p. 178, 183), and reflects well the aspect of reuse of data imbued in FAIR. However, on the other hand, the shareability of (Indigenous) data to other researchers and wider society raises concerns from the perspective of local communities. Traditional knowledge, sometimes closely linked to (sacred) lands, is an example where sharing is not always desirable. Such tensions need to be addressed in order to find sustainable ways of sharing Indigenous research, as this article hopes to contribute.

Colonial relations in the recent past have left traces in how research is perceived today. Moreover, colonialism is still present in several domains, such as power relations and decisions, well-illustrated by the issue of the exploitation of Sámi land motivated by national interests but threatening traditional Sámi life. Therefore, the field of Indigenous research calls for and necessitates a careful, reflexive, and well thought-out approach to the researcher's positionality and relationship to the data, subjects, and lands etc. that are involved in and concerned with the research. In a Sámi context, the lack of return of knowledge, of information about sources, combined with assimilatory and paternalistic ideologies have influenced earlier research. Such research also resulted in the exoticization of Sámi culture that was given “an object status for scientists outside the cultural fellowship” (Lehtola, 2017, p. 163).

Research on data sovereignty in a Sámi context is scarce, but has recently received more attention as ethical guidelines for Sámi research are under development in Norway, Finland and, to some extent, Sweden [Sámediggi (Sámi Parliament of Finland), 2016; Kvernmo et al., 2018; Sámiid Riikkasearvi, 2019]. Axelsson and Storm Mienna (2020), conducting a project in Swedish Sápmi, have approached the topic of data governance in focus group interviews. They observe how the fact that misuse and inappropriate use of data in the past is still very present in the minds of the participants when considering possible risks when giving access to data (in this case, related to health).

In an international research context, Indigenous data sovereignty, i.e., “the right of Indigenous peoples to govern the collection, ownership, and application of data about Indigenous communities, peoples, lands, and resources” (Rainie et al., 2019, p. 301) is discussed in relation to self-determination and in an effort to restore trust between academia and Indigenous communities. In this process, Indigenous scholars play a key role in redefining ethically valid and respectful ways to conduct research, in a culturally-sensitive manner.

One example of one such effort is the development of the CARE principles as a complement to the FAIR principles. CARE principles encourage “open and other data movements to consider both people and purpose in their advocacy and pursuits”.^2^ Essentially, the CARE principles—standing for Collective benefits, Authority to control, Responsibility, and Ethics—are “people and purpose-oriented” and have to be understood in relation to the values promoted by United Nations and the right to self-governance. The CARE principles were developed and established in 2018 by the Global Indigenous Data Governance network (GIDA)^3^ for the purpose of increasing accessibility and shareability of data (in line with open science principles), but also to assess Indigenous Peoples' control over their data (in line with the UNDRIP). The purpose is also to increase the value of Indigenous data for the Indigenous communities, i.e., support the use of data in “ways that are grounded in Indigenous worldviews”. The CARE principles strive to empower the communities and their decision-making power in the collection, use and curation of data and metadata and for connecting to principles from Indigenous research methodologies such as relationships built on respect and reciprocity, and Indigenous ethical frameworks. In June 2021, a GIDA-Sápmi network was established with the goal of adapting and operationalizing the CARE-principles in the context of Sápmi, and to advance the discussion of Sámi data governance (Research Data Alliance International Indigenous Data Sovereignty Interest Group, 2019).

The emergence of the CARE principles, and the articulated need to complement the FAIR principles, illustrate a recurring set of problems when applying standardized principles to minority and Indigenous research. The establishment of standards seldom succeed at including the diversity of interests and perspectives. Discussions about ethical pluralism are another illustration of such a set of problems. As it has been demonstrated in previous research, principally in the field of research ethics, “most of the discussion and reflection on digital media ethics took place primarily within Western countries” (Ess, 2009, p. 168). This implies that perspectives and ways of thinking from Western cultural and linguistic contexts have been used when setting ethical standards. Although there is an increasing body of literature addressing these dimensions (for example, see George et al., 2020), more is needed when it comes to Indigenous research.

In addition to the ongoing work of implementing the CARE principles, Indigenous scholars have been developing common principles aimed at guiding researchers in planning, conducting, and disseminating research. These are summarized in the literature as the 4 R's of Indigenous research, that is, the four key principles of Reciprocity, Respect, Responsibility and Relevance (Kirkness and Barnhardt, 2001; Reid and Taylor, 2011), sometimes also formulated as Relational accountability, Respectful representation, Reciprocal appropriation, and Rights and regulation (see for instance Louis, 2007; Chilisa, 2012). Approaching open science with these principles in mind can shed light on issues and possibilities when working with Indigenous data, and on ethical matters in this context.

Establishing good relationships in research is central for successful research projects–that is true when it comes to collaborations as well as the collection and production of data, etc. Qualitative methods such as interviews are clear illustrations of this. In minority and Indigenous research, good relationships between researchers and local communities are a prerequisite sine qua non. The importance of establishing sustainable relationships is expressed through the principles of *Respect* and *Reciprocity* (Kirkness and Barnhardt, 2001).

The principle of *Responsibility* is yet another guiding notion that might help us toward finding solutions for sustainable ways to deal with the principles of open science. Research ethics (regardless of the research discipline) call for responsibility and remind us that “increased vulnerability requires increased responsibility” (cf. e.g., adults in relation to children) (Ess, 2009, p. 12). The term “vulnerability” in relation to particular groups in society can be questioned, but there is an important aspect that needs to be addressed, i.e., the question of if, and how, certain people around the researcher are more “vulnerable” in the (research) situation they create. Such a situation might be when recording an interview, when sharing this interview with others, when collecting data published on online platforms, etc. In the case of Indigenous research, the researcher's responsibility comprises several dimensions (Kovach, 2009). It is about accountable responsibility (see for instance Ellis and Earley, 2006; Chilisa, 2012): the researcher should be accountable for all parts of the research. It is also about ethical responsibility (Chilisa, 2012, p. 18), not only complying with ethics, but also contributing to implementing and improving ethical guidelines and principles in line with Indigenous protocols.^4^

Beyond the responsibility of “do no harm” (a core ethical standpoint), there is also an urge to do relevant research. This leads us to the fourth R of Indigenous methodologies: *Relevance*. This principle reminds us that research can be interpreted as a form of appropriation and therefore should have relevance for the research participants (and not only researchers). This principle can guide us in the process of sharing data, for instance, in relation to identified data gaps and specific needs of the communities.

## Materials and methods

In the context of a project about data management support for researchers in the humanities at our university, I conducted interviews with researchers from various fields in the late 2022–early 2023. One goal was to identify what kind of support our research infrastructure within Digital Humanities could offer. The questions in the interviews covered topics such as what kind of data is used, how it is selected; what considerations are made for a project regarding documentation, metadata, and accessibility; how anonymity and confidentiality are addressed; and what assistance the scholar has access to in term of legal, practical, and technological support. The interviews also encouraged participants to share reflections about open science and FAIR-principles in relation to the scholar's specific project, data and publications. Particularly, in an effort to identify the tensions at play between open science principles and Indigenous research, scholars active in the area of Sámi research (five interviews) were asked specific questions about potential challenges, risks, and benefits with open science in the case of Sámi and Indigenous data.

Additionally, policy documents published by Sámi authorities (*Sámediggi* (Sámi parliaments of Norway, Sweden and Finland), *Sámiid Riikkasearvi* [the National Union of the Sámi People in Sweden) and *Sámirađđi* (the Sámi Council)] have been examined in order to include discussions about data and ethics at a policy level. In 2019, *Sámiid Riikkasearvi* published a policy for collaboration between researchers and the Sámi community. Although it is not explicitly framed as ethical guidelines, the underpinnings are clearly based on the value of relationships, respect, and power of influence. The text also includes questions about research data. Relevant documents by the *Sámediggi* of the Nordic countries include one about traditional knowledge (Sámediggi of Sweden, 2010), one about consultation and consent (Sámediggi of Finland, 2019) and one suggesting guidelines for guarantying the ethical basis of the health research (Sámediggi of Norge, 2020). The report entitled “Working toward ethical guidelines for research involving the Sámi” (2021) by Áslat Holmberg, the Sámi Council is a recent report that summarizes the status of recent and ongoing works on ethical guidelines in Norway, Sweden, and Finland, and suggests a path toward the development of common Sámi guidelines.

A content analysis of the policy documents and the interviews was conducted in the purpose to identify key themes and capture recurring patterns across and within the data sets. Problems, mismatches, or even clashes when researchers address the recommendations of open science, not least in relation to data management, were recurring points of discussion along the interviews. The scholars interviewed have various disciplinary backgrounds and various ways to relate to Indigenous methodologies–central for some, peripheral for other. The general content of policy documents, or the discourses these are inscribed into (such as a need for closer collaboration between researchers and local communities) were referred to in the interviews, implicitly, or explicitly. The variety of disciplinary backgrounds of the interviewees implies that the degree of tensions and importance of some issues related to open science, are approached differently. The interviewed scholars also work with a variety of data and with distinct groups (health data, school children etc.) and the diversity of kind of materials is also discussed in the policy documents. Despite the discrepancy, recurring topics commonly addressed in the interviews and policy documents could be quickly identified through the content analysis: ownership, control, sensitivity and accessibility of data, the consequences of experienced limitations, the role of the historical context, and community-groundedness.

## Researchers' dilemmas

The issue of *ownership of data* is mentioned by the interviewees as a “difficult” or “impossible” question. Universities, as employers and formal owners of research projects (in a Swedish academic context, similarly to other national contexts), own the data collected or co-created within research projects conducted by their employees. As an interviewee observes, this implies that “we cannot guarantee our participants that what they share with us will not be used by others, in other projects or in other purposes” (interview 1). In Indigenous research, the importance of reciprocal relationships and trust means that the relationship between a participant (for instance, who contributes with an interview) and a researcher is key. “It is about trust: people would probably not accept to participate or contribute to our projects if we were to share our research data” (interview 3), observes one of the interviewees. Another scholar comments that there is a risk that participants declare that they are not interested in contributing to a project if they cannot know how their data might be used in the future (interview 4). The fact that the ownership of the data rests in the hands of a third party (a national authority such as a university) challenges this relationship and might affect a person's willingness to share information and participate in a project.

In their policy document about project collaborations (2019), *Sámiid Riikkasearvi* advises “[r]esearchers interested in starting collaborations with Sámiid Riikkasearvi […] to think through and answer a number of questions prior to contacting Sámiid Riikkasearvi”. Several of these questions explicitly address the issue of ownership, such as “Who owns the research?” and “What happens to the research data—who owns it?”. They also ask for a consent document that should answer to (among other things) the question of how research data will be handled “now and in the future”.

It is, however, at this point difficult to identify how and where ownership could be transferred. One of the interviewed scholars explains that

It would preferable if it could be owned as closely as possible to those who have been responsible for the production of the data. That it is not in the central archives in the capitals. It should be available to them [the participants], close to the participants. Somewhere fundamentally, it is their knowledge and stories. (interview 5)

Another interviewee expresses that they feel it is “safer that we have our material here [at the university] than it would be at, for example, *Sámediggi*. There are currently no procedures there for storing it safely” (interview 3). Similarly, *Sámirađđi* observes that “many Sámi communities lack representative bodies with the capacity and mandate to deal with issues related to á*rbediehtu* [traditional knowledge]” (Sámirađđi, 2021, p. 1).

In practice, the researchers do not necessarily see this as a problem. An interviewee comments that

In the end, I think that many people think that the universities are the logical administrators, and from my point of view it is logical. I think the university should manage this kind of data [sensitive personal data]. It is perfectly reasonable. Then you can have a steering group that protects the material, so to speak, that has strong Sami expertise, for example. (Interview 2)

Further, the question of *control of data* is addressed in the interviews as a priority. Researchers emphasize the importance of having control of the data collected during a project. For one of them, it has to do with a risk of misinterpretation:

Sharing data can allow to conduct new analyses, or conduct other analyses based on other methods. That's very good. The problem is how to deal with sensitive data. There may be a risk that the data is taken out of context. Within indigenous research, I, as a researcher have a specific research position, and the data is created in a specific context, and there is a risk that the data can be misinterpreted in a different context by another researcher in a different position. And it can be, it has been, harmful to Indigenous groups. (Interview 5)

The interviewed scholars mention different strategies for ensuring that they as project leaders can keep control of the data and limit the risks that the data can be lost, corrupted, or leaked. Outsourcing interviews for professional transcription is to be considered carefully, mention one interviewee (interview 4). Storage is another phase in the data cycle that requires careful consideration, and the interviewees mention the choice of using only local devices or storing hard copies. A strict selection of limited actors who have access to the data, and the choice not to share the data, are mentioned as other strategies. This priority is motivated both in relation to the participants—to guarantee that the data they shared within a project do not risk leaking—and in relation to security aspects and the risk that a shared folder or too generous access might imply that the data gets in the wrong hands.

These strategies and choices comply with the people and purpose-oriented approach proposed by the CARE-principles. On a policy level, this is supported by the *Sámirađđi*. In their aspirations about the work on Sámi research ethics in the future, the report mentions the role of GIDA-Sápmi. It highlights the intertwinement of the processes toward the development of Sámi research ethical guidelines with the ongoing work focusing on data sovereignty and data management (Sámirađđi, 2021, p. 10).

Another recurring topic given attention to in the interviews is about the *sensitivity of data*. Ethnic origin, for instance (along with political opinion, religion, trade union membership, sexual orientation, or genetic, biometric or health data) is classified as sensitive personal data according to the General Data Protection Regulation (GDPR). Beyond the legal framework defining personal data and sensitive personal data, the researchers problematize the kind and degree of sensitivity in their specific data in relation to their own ethical standards and in relation to those concerned with the research. In some cases, the ethnicity of the participants is what makes the data sensitive. In other cases, the data itself requires levels of consideration, for instance in the case of health-related data.

Genomic articulations of indigeneity (see for instance TallBear, 2013) have been shown to have critical implications in identity-making processes for and by Indigenous groups. This also echoes with discourses from racial biology and phrenology that are still present in the mind of Indigenous and minority groups in the Nordic countries. The same interviewee comments that “[in] some contexts, you may not just be able to see data as any data; especially in Indigenous contexts”. (Interview 2)

Consequently, the question of *accessibility of data* becomes a delicate one. The interviewed researchers stress the importance of giving back to the community and the participants, i.e., sharing data and results in a way that makes sense ethically and culturally—and in line with Indigenous methodologies. This is particularly true on an individual level in the case of health data, comments one interviewee, or on the collective level of the community in the case of á*rbediehtu*, stresses another one.

The importance of making data shareable and accessible is not given, and it is actually not mentioned by the interviewed researchers as a priority. One interviewee mentions frustration about the difficulty to find a safe and secure way to share data with other members of the project, but sharing data with other scholars not included in the project is not on the agenda. One interviewee reflects about this aspect particularly in relation to Indigenous research compared with other fields of research:

The goals must be clear. There must be a different form of security, respect and responsibility for this data and how it is used. (interview 3)

One of the interviewees shares their concerns with the *risk of some research areas being threatened by the standards* required for data management and ethics. While they express no doubt about the importance of developing and applying regulations and principles appropriate to Indigenous research, they observe the consequences of the difficulty to navigate among these: “you need to be an expert in all aspects—technical aspects, legislation, your discipline etc.” (interview 1). They mention a feeling of “unfairness” when some areas of research are subject to principles and guidelines, while other researchers and disciplines do not need ethical vetting and can easily find straight forward solutions for data management. They see a risk there and have experienced how colleagues have chosen not to conduct important research, because the type and level of sensitivity of data makes the process uncertain and very complicated. One problem clearly identified is that support is not in place. “Already early in the process, you find out that there is a lack of support and answers” (interview 1), they explain when looking back at their experience in searching for solutions about how and where to store large amounts of sensitive data.

Another interviewee reflects on the risk that certain methodologies might not be possible to apply in a context where they cannot guarantee who would have access to the data collected in their project, for instance when taking advantage of ways of communication and knowledge exchange more appropriate in certain Indigenous contexts than formal interviews. “This means that you cannot work with the kind of conversations that are about building trust”, reflects an interviewee (interview 4), thinking of occasions when participants can mention things out of topic, sometimes about someone else, without any direct relevance for the project itself.

The interviews indicate that Sámi research is facing challenges that must be solved urgently, since the consequences of this present situation for younger researchers especially, but also for research and knowledge production in general, might be that important aspects of Sámi life will be avoided in future research. Discussions about the *importance of the historical context* are also revealed in the interviews when considering the implications of using various kind of data. One interviewee comments how “that thing with the Institute of Racial Biology still is terribly sensitive” (interview 2) and another one pertinently observes that “sensitive data is sensitive data, regardless of when it was collected” (interview 3). Misuses in historical times (including recent history, primarily the Swedish Institute of Racial Biology) creates the need for careful consideration of data. It requires also knowledge about the context, reflect one interviewee:

There is also a risk of misinterpretation, that the data is interpreted by researchers who do not have knowledge of the history of the area the data comes from. There is always a risk of misinterpretation, distortion, or exotification. That is common, that it becomes exotic. (Interview 5)

Moreover, as another interviewee observes, research in earlier periods of time did not have to comply with the ethical guidelines we have today, and we often lack information about the context in which the material was collected or created. Another one comments the risk of forgetting the “ethical lens” when working with historical data, despite the fact that part of the context of collection can be problematic (interview 4). This implies that such data might be more problematic to use than contemporary data from a project that has received ethical approval and was conducted by researchers complying with the guidelines and recommendations in Indigenous research.

A recurring aspect when discussing ethical issues with the interviewees is the importance given to *community-groundedness*. It is something expected in the context of Indigenous research, but there is an interesting discrepancy between how various kinds of expertise are invoked. In interviews with scholars active in Sámi research, less focus is put on legal aspects than in interviews with researchers in other disciplines. Legal and ethical frameworks are considered as a minimum, and community experts are given great importance. For instance, one interviewee refers on several occasions to the results of interviews as the basis for ethical choices made later in the project, e.g., in the design of a questionnaire, in dealing with collected data, and in communicating the results of the project.

The dissemination of research results as a way of reporting, a form of restitution of knowledge and of giving back to the communities involved in the research is a key principle in Indigenous methodologies. To borrow Linda Tuhiwai Smith words, sharing “is a responsibility of research” (Tuhiwai Smith, 2008, p. 161). Denzin et al. underscore, for instance, the fact that “[Indigenous persons], not Western scholars, should have first access to research findings and control over the distribution of knowledge” (Denzin et al., 2008, p. 2). Previous research has shown that scholars in Sámi studies apply a variety of strategies and channels for communication of research, such as videos shared on social media, conferences addressing various groups of stakeholders and adapted to their specific interests, educational programmes, non-academic publications such as reports in the national language, articles in newspapers and participation at events organized by the community (Cocq, 2022).

In the policy for research collaborations published by the Sámiid Riikkasearvi, it is stated that “information about the project should be disseminated to the research participants—a well-defined plan shall be created for how this shall be implemented.” The document also asks potential collaborators to address the question of how researchers will “'give back' to Sámiid Riikkasearvi and other research participants”. It is, in fact, a priority mentioned in the interviews about Sámi research in particular. One interviewee underscores the central role played by the Indigenous communities they work with in the research process, while the research community as a partner for dialog and for dissemination is secondary. “I feel that the reporting requirement is super important”, says another one, for instance. This implies finding modes of sharing results in a culturally appropriate way. In other words, ensuring that research results are accessible is a given. The main question in this case is rather how to share research results in a manner that is relevant for a local community for instance. Conference papers and articles published in open access international journals are appropriate when we want to make our research accessible to the research community. When we have other audiences in mind, we need to choose more appropriate ways to share and disseminate our research. This can involve the choice of communication channels, the language or languages used, the format (text, public meeting, conversations, digital platform etc.).

## Discussion

The need to maintain sustainable relationships, ensure trust and respect—valid in relation to any participant in research—has, in the context of Sámi research, to be understood in the light of history. This is similar to what have been observed in other Indigenous settings, e.g., by Bronwyn Carlson:

And trust […] is complicated. For non-Indigenous people, the intersections of critical studies, technology, culture, and society look vastly different than for the Indigenous populations who also factor in colonial history and technology, and the ways in which identities are inherently linked to place, genealogy, kinship, and language (Rowe, 2021).

Discussions about the control of data are closely related to historical aspects in Sámi research—or, more explicitly, to the legacy of colonialism and how it came to manifest when collecting not only intangible traditional knowledge but also, and not least, artifacts (from handicraft to sacred objects), ancestors (human remains), biometrical data, and photographs from exposed bodies for racial classification. In most cases, the types of data collected during a time of discriminating ideologies toward minorities are currently possessed by museums, and restitution of ancestral remains has taken place only in a few and rare occasions. As shown in previous research, and as the interviewees confirmed based on their experiences, history is very much present and of immediate interest for many Sámi, across several generations. In this context, it is not surprising that the collection of digital data raises concerns, and that contemporary researchers and allies struggle to find ways to secure the control of data. In light of the principles of Respect and Reciprocity, the question of what happens with the data after a project's completion, is crucial to address. Once the publication and dissemination phase of a research project is over, the archiving of data re-actualizes the issue of ownership and control of data. The efforts articulated by the CARE principles, more particularly about Collective benefits and Authority to control, address this issue by proposing a repositioning from research institutions to local communities regarding the administration of research data.

United Nations clearly states that Indigenous Peoples

have the right to maintain, control, protect and develop their intellectual property over […] cultural heritage, traditional knowledge, and traditional cultural expressions (UNDRIP 2007, article 31).

The implementation of these rights poses however some challenges, as for instance *Sámirađđi* observes:

in some instances, there might be a contradiction between laws and guidelines. Standards set in international law on Indigenous Peoples' right to self-determination and intellectual property rights are not fully adopted into national legislations in the 4 countries which overlap with Sápmi (Sámirađđi, 2021, p. 7).

The use of digital data illustrates how these contradictions concretize in research. The topic is timely and sensitive, and data use as a form of colonialism is discussed to an increasing extent in research literature. Couldry and Mejias' influential work (Couldry and Mejias, 2019a,b) shows for instance how the ways data are used today can be analyzed as a form of coloniality that reinforces power inequalities inherited. They describe data use in term of “colonialism,” in that “[w]hat is going on with data [is] a form of fundamental appropriation (Greene and Joseph, 2015; Thatcher et al., 2016), or extraction (Mezzadra and Neilson, 2017) of resources” (Couldry and Mejias, 2019b, p. 338).

In the contemporary context of Sámi research, many actors, from national authorities to research institutions, community-driven local organizations and cultural institutions, compose a complex net of actors, where colonial practices cannot easily be connected to a specific agent, but rather imbue some practices taking place at different levels. Therefore, the concept of data colonialism can be nuanced and revised in the light of another concept: surveillance culture, proposed by Lyon (2018). This has been observed elsewhere and described as “soft surveillance” (e.g., Marx, 2005), i.e., a form of surveillance not merely something that states, authorities etc. impose on us, but rather something that we do, submit to, participate in. The role of research, researchers and their institutions in this process needs to be examined. Changes in research practices are taking place, partly because of an agenda toward the implementation of open science. The potential Reuse (as the R in FAIR) of shared data raises the question of monitoring and control of data. Therefore, the addition of the principle of Authority to control (as the A in CARE) is a valuable step toward establishing trust and reliability between academics and community members.

Taking into consideration these tensions, we need to reflect upon the implications of these changes. Furthermore, the multiplicity of actors involved in the production, collection, and use of data means that collective efforts are required—on a political level, on the level of researchers and their institutions, and on the level of organizations, groups, and individuals—at best, through collaboration between these actors.

### Suggesting solutions

The question of ownership and control of data in Swedish Sápmi highlights the larger structural challenge of representation of the Sámi communities: there are no Sámi universities or other educational bodies that are clearly community-driven. *Sámediggi*, as a political authority, has no specific (formal or informal) mandate for coordinating research or, consequently, take the responsibility of control over research data. The interviews conducted by Axelsson and Storm Mienna show how participants “thought that data should be owned and managed by Sámi themselves, but recognized that no such system was currently in place to make that happen” (Axelsson and Storm Mienna, 2020, p. 105).” Researchers express in the interviews that it would be appropriate to have the data close to the communities, but also that the university is a logical and practical solution since there are regulations, routines, and expertise for taking responsibility in the matter. The concerns of the researchers are about how to ensure trust and strengthen relationships between academic institutions and community members. Here, the efforts of researchers active in Sámi and Indigenous research are clear and well-articulated. The structural problem of the inheritance of misuse of data in research, and an underlying risk for ideologies that counter principles of Indigenous methodologies, are issues that project leaders and research groups cannot easily influence to make a change. The responsibility of universities as employers and as research institutions is critical, and their readiness for working actively in building sustainable relationships with actors in Sápmi will be crucial for the future development of open science. As the interviewees make clear, the situation is so diverse when it comes to the multidisciplinary characters of research projects, to the multiple actors and communities in Sápmi and the diverse interests they represent, that it would difficult, and, according to one interviewee, even “inadequate” to decide upon a standard solution for all projects in Sámi research. A case-by-case based approach and flexible frameworks are to be preferred, given that a common baseline can be established (such as the CARE principles). The development of protocols by universities and research institutions together with key Sámi institutions could, for instance, establish a list of criteria to help researchers navigate their decisions about what steps to take into consideration when dealing with data from Sámi individuals and knowledge. Depending on the nature and degree of sensitivity of the data, such protocols would refer to policies appropriate for the specific research case.

The interviews give a clear picture of the difficulties researchers encounter in planning projects due to the lack of, or the scarcity of, access to legal and technical expertise. In some cases, this might lead to the choice to give up on a research idea. In other cases, the time dedicated to finding solutions results in less time to dedicate to research itself—with direct consequences in term of funding and career development. While many initiatives are taking place for establishing routines for the implementation of open science and for ensuring adequate data management, resources for researchers working with sensitive data are less discussed. An aspect where universities (and actors within universities) can act in order to facilitate an ethical implementation of open science principles and data management in Sámi research is in allocating resources for support. Such resources would include personnel with specific knowledge and specific standards for data curation. Additional research time for research projects (where funding is often translated as a number of weeks, months, or years) would also enable project leaders to consider collecting and curating relevant data, and thereby lowering the risk that certain kinds of research or methodology are avoided or opted out.

Research on data governance and the interviews I conducted in the context of Sámi research underscore the role of politicians in the process toward finding adequate ways for the implementation of open science. One interviewee recommends that “we can wait a bit, there is a lot that needs to happen in Sámi society” (interview 2) in relation to recent initiatives taken by Sámi institutions and ongoing discussions about the topic (e.g., see Sámirađđi, 2021). In sum, if the work needed to be performed by researchers and their research institutions is to be successful, it should be community-driven and develop in dialog with Sámi institutions, organizations, and their representatives. Such a development has proved to be highly valuable in other Indigenous contexts (e.g., see FNIGC^5^ for the example of Canada and Hudson et al., 2010 in a Maori context). Joint initiatives are taking place in Sápmi, for instance, in relation to the establishment of the GIDA-Sápmi network (and related seminars and conferences), and in the process of developing ethical guidelines.

The principle of informed consent is a central principle in ethics. In Indigenous research, FPIC, i.e., Free, Prior, and Informed Consent is articulated as a way of ensuring that Indigenous communities are consulted before the development and implementation of projects and initiatives that could impact the community or the land. The procedure of obtaining individual consent prior to interviews, for instance, is well-established, and is required for instance be Sámiid Riikkasearvi (2019). In addition, collective consent has been suggested as a way to way for accessing larger sets of data, or data that concern a larger group. This has already been implemented in many Indigenous contexts and is becoming a key principle of research policies and ethical guidelines in Indigenous research. In Norway, a recent implementation of this principle has been established for regulating health data about Sámi individuals (Sámediggi of Norge, 2020). Such initiatives still need to be formalized elsewhere in Sápmi, and an increased dialog between national, authorities on ethics and Sámi organizations would be beneficial for research and research processes. The inclusion of Sámi scholars and/or representatives in national, ethical research boards would be a first, necessary, and not far-fetched step to take.

While the main focus of this article, led by the content of the interviews, is on the issues of ownership, control, and shareability of data, it is also important to remember that the processing and categorizing of data—when being shared, for instance—also present challenges. In an international context, examples of the application of Indigenous methodologies to digital data highlight various modes of access to data. For instance, the platform Mukurtu^6^ hosts projects related to Indigenous cultural heritage. Designers behind the platform have, together with Indigenous groups, developed cultural protocols for presenting and regulating access to the data (Christen, 2011; Senier, 2014; Montenegro, 2019). Mukurtu requires membership for full access and gives the communities control over the data curated by using traditional heritage labels and defining and implementing cultural protocols.

In a Sámi context, initiatives have been taken in order to curate and protect access to archival data, e.g., a portal developed by the project Digital Access to the Sámi Heritage Archives,^7^ giving access to archival materials stored in disparate museums and archives. The AIDA^8^ project (Arctic Indigenous Design Archives) is another example, as an archive for *duojár* (sámi crafters). These projects work for establishing Sámi ethical guidelines for dealing with archive materials, addressing, for instance, the implementation of archival legislation in relation to Indigenous ethics, developing ethical guidelines that take into account different knowledge systems, preventing the derogatory, culturally or otherwise offensive use of cultural heritage materials. These examples are welcome efforts for researchers and teachers, as they problematize and provide ethically valid ways to access valuable materials about cultural heritage and traditional knowledge.

## Conclusions

The current development toward a rapid digitalization of research practices and an increased access to digital data raise concerns. Couldry and Mejias problematize data relations as “new types of human relations that give corporations a comprehensive view of our sociality, enabling human life to become an input or a resource for capitalism” (2019a, p. 85). As researchers, we need to address the risk (and, indeed, the practice) of exploitation of human life through data. When collecting and producing data, we should do our best to avoid contributing to such a form of exploitation of human life. In other words, we need to establish sustainable data relations in research, i.e., find ways to make the use of data in research based on principles of respect and reciprocity.

Research—in relation to ethics and social sustainability—is an arena where tensions between various agendas becomes obvious. This is illustrated in this article by researchers' dilemmas when working with open science and the advancement of Sámi research. This article also hopes to contribute to further discussions about how to develop research procedures that respect Indigenous research sovereignty.

Couldry and Mejias remind us that the transformation needed is social, not technical (Couldry and Mejias, 2019a, p. 214–215) if we want to resist processes of data colonialism that maintain inequalities. It is a prioritized issue on the agenda of Indigenous researchers and allies. Efforts toward ethically valid and cultural-sensitive modes of data use are taking shape in Indigenous research, calling for an increased awareness about the topic. In the context of Sámi research, taking into account the Indigenous principles of reciprocity, relationships, respect and relevance in such a transformation is essential.

## Data availability statement

The datasets presented in this article are not readily available but may be provided upon request following the consent of the participants. Requests to access the datasets should be directed to coppelie.cocq@umu.se.

## Ethics statement

Ethical review and approval was not required for the study on human participants in accordance with the local legislation and institutional requirements. The patients/participants provided their informed consent to participate in this study.

## Author contributions

The author confirms being the sole contributor of this work and has approved it for publication.

## Footnotes

^1^For instance Sámi culture and Sámi languages are taught at several universities in the US.

^2^ https://static1.squarespace.com/static/5d3799de845604000199cd24/t/5da9f4479ecab221ce848fb2/1571419335217/CARE+Principles_One+Pagers+FINAL_Oct_17_2019.pdf

^3^ https://www.gida-global.org

^4^See for instance https://aiatsis.gov.au/research/ethical-research/code-ethics.

^5^ https://fnigc.ca/wp-content/uploads/2022/10/FNIGC_Brochure_20220927_web.pdf

^6^ https://mukurtu.org

^7^ https://digisamiarchives.com

^8^ https://arkisto.fi/aida
